# The Sexual Function Evaluation Questionnaire (SFEQ) to Evaluate Effectiveness of Treatment for Sexual Difficulties: Development and Validation in a Clinical Sample

**DOI:** 10.1080/00224499.2021.1986800

**Published:** 2021-11-15

**Authors:** Kirstin R Mitchell, Karen Gurney, Kareena McAloney-Kocaman, Caitlin Kiddy, Alison Parkes

**Affiliations:** a MRC/CSO Social and Public Health Sciences Unit, University of Glasgow; b Sexual Health and HIV Care, Chelsea and Westminster NHS Trust; c Department of Psychology, Glasgow Caledonian University; d Clinical Health Psychology (Psychological Medicine) - Chelsea & Westminster Hospital, CNWL NHS Foundation Trust

## Abstract

Progress toward establishing the effectiveness of biopsychosocial treatment for patients with sexual problems is limited by the lack of brief measurement tools assessing change across various domains of the treatment model. We developed and psychometrically validated a new clinical evaluation tool, the Sexual Function Evaluation Questionnaire (SFEQ) to meet this gap. The SFEQ combines into a single scale the best performing items from two instruments that were piloted in a UK sexual problems clinic (*n* = 486): the Natsal-SF Clinical Version and the National Sexual Outcomes Group 1 measure. Internal construct validity evidence from exploratory and confirmatory factor analyses supported a 16-item measure consisting of one overarching dimension of overall sexual function distributed along four subscales: problem distress, partner relationship, sex life, and sexual confidence. The measure had satisfactory configural, metric, and scalar invariance over time and across groups based on gender, ethnicity, and age. Correlations with patient depression and anxiety demonstrated external validity. Change in scores over the course of therapy varied as predicted, with greater improvement in younger patients and in areas more amenable to change via therapy (sexual confidence and problem distress). The SFEQ is a brief clinical tool with the potential to assess sexual function and evaluate the effectiveness of biopsychosocial treatment programs.

Treatment of sexual difficulties often requires addressing complex causes rooted in psychological, relational, and socio-cultural spheres (McCabe et al., 2010). The biopsychosocial model takes account of these wide-ranging influences on sexual dysfunction (Brotto et al., 2016) and is thus recommended as a framework for understanding and treating sexual problems (McCabe et al., 2010; Thomas & Thurston, 2016). In recognizing the multiple levels of influence on the experience of disease, the biopsychosocial model represents a philosophy of clinical care that recognizes the importance of patient subjective experience in accurate diagnosis and effective treatment (Borrell-Carrió et al. 2004). Biopsychosocial treatment approaches typically combine psychological approaches (such as mindfulness training or cognitive-behavioral therapy) with pharmacological treatment (for example, McCabe & Althof, 2014; Schmidt et al., 2014), and they rely on open patient–clinician relationships and intuitive diagnostic skills (Borrell-Carrió et al., 2004).

Despite their promise, clear biopsychosocial treatment models and algorithms are still in development, and there are practical challenges in ensuring interdisciplinary training and collaboration across clinical providers (Berry & Berry, 2013; Brotto et al., 2017; Pyke & Clayton, 2015). One issue with biopsychosocial treatment is the lack of agreement about what constitutes a successful outcome, given the primacy given to patient assessment of the problem (Leiblum, 2006; Philips, 2009). Another issue is that variation in approaches across practitioners, and the importance of dynamics between clinician and patient, makes it difficult to identify and replicate the “effective ingredients” of the approach.

A third issue is the limited number of valid and appropriate instruments that could be employed to demonstrate effectiveness (Pyke & Clayton, 2015; Tabatabaie, 2014). Widely used “gold standard” instruments such as the International Index of Erectile Function (IIEF; for men; R. C. Rosen et al., 1997) and the Female Sexual Function Index (FSFI for women; R. Rosen et al., 2000), are too narrowly focused. For instance, both assume vaginal penetration and have limited questions on the relationship or on patient assessment of “bother.” Another measure widely used in clinical settings – The Changes in Sexual Functioning Questionnaire - Short Form (CSFQ-14) (Keller et al., 2006) – is brief and less focused on physiological response but does not assess the sexual relationship and does not include items on sexual confidence that is often a target of sex therapy (Leiblum, 2006).

Indeed, while many measures of sexual (dys)function exist, few are designed to offer a holistic assessment in clinical settings. Limitations of existing measures include weak psychometric properties, length (too long for clinical use), and applicability to only specific groups, such as women only, men only, those in longer term relationships, or with opposite-sex partners (Arrington et al., 2004; Daker-White, 2002). A review of potential sex therapy outcome measures focused on those that were potentially sensitive to change; applicable across a wide range of male and female sexual and relational problems; had strong psychometric properties; and were easy to administer and complete (Tabatabaie, 2014). Although several measures were identified in the review as meeting these criteria, none spanned across all dimensions of sexual problems, sexual relationships, and general quality of sex life (Tabatabaie, 2014). There is thus a key gap in the field for a brief clinical evaluation tool that can assess change in sexual function holistically, that is: sexual difficulties; relational aspects of sexual function; and patient feelings about problems (e.g., satisfaction, distress, and confidence).

In order to assess the possibilities for a new clinical evaluation tool capturing change in different domains of the biopsychosocial model, we carried out a psychometric evaluation of two instruments that were piloted in a sexual problems clinic in the UK: the Natsal-SF Clinical Version (NSFC) and NSOG1. The Natsal-SF-Clinical Version was adapted from the Natsal-SF measure, a brief (17-item) bespoke measure for the third British National Survey of Sexual Attitudes and Lifestyles (Natsal 3). The Natsal-SF was designed for community health surveys to provide an annual population prevalence estimate of sexual function (Mitchell et al., 2012). It measures a general construct of sexual function, with three specific factors measuring sexual problems, sexual relationship, and appraisal of sex life. The Natsal-SF has good discriminant validity (odds ratio [OR] 2.667 for clinical group), acceptable test–retest reliability (*r* = 0.72), and good model fit (Comparative Fit Index 0.963; Tucker Lewis Index 0.951; and Root Mean Square Error of Approximation 0.064; Mitchell et al., 2012). However, the Natsal-SF is unsuitable for clinical use because of its 1-year reporting period and lack of detailed information on the severity and distress of sexual problems. In 2014, the first author was requested to adapt the Natsal-SF for clinical use. In addition to small tweaks to instructions and wording, the three significant adaptations were as follows: to reduce the timeframe for reporting from past year to past month; to include items on distress about problems; and to remove the final item on help-seeking (Mitchell et al., 2016). The Natsal-SF clinical (NSFQ) was then piloted in a single sexual problems service in London led by KG. In parallel, a group of cross-specialty clinicians formed the UK National Sexual Outcomes Group (NSOG), with a remit to design a brief evaluation measure for use in their services. Committed to biopsychosocial treatment of sexual problems, the group was motivated to address a perceived gap in suitable measures (i.e. brief and holistic). The first version they developed (NSOG1) – a brief 8-item measure – was designed via discussion between group members and focused on self-appraisal in terms of confidence, satisfaction, distress, quality of sex life and importance of sex. The measure was not psychometrically validated but was nonetheless piloted by the group across several UK sexual problems services, including alongside the Natsal-SF in the service led by KG. We originally set out to validate each measure separately. It soon became clear that due to limitations inherent in each measure and non-overlap in topic coverage, a single combined measure would be stronger and have wider applicability. This study investigated the internal and external validity of the combined instrument (the SFEQ; male and female versions) and its responsiveness to change over a course of therapy sessions in a sexual problem clinic setting.

## Method

### Sample

The study used fully anonymized records from 486 patients at the Sexual Problems Assessment and Treatment Service, Central and North West London NHS Foundation Trust collected between August 2015 and December 2018. The model of this service included assessment (and possible treatment) by both a doctor trained in psychosexual medicine and psychosexual therapy by a clinical psychologist. Patients supplied information on the NATSAL-SF Clinical and/or NSOG prior to their initial patient assessment, and on repeated occasions during their involvement with the service. The full sample comprised 486 patients (45.9% female) who supplied data at Time 1 (initial assessment; see Table 3). This was a sample for the analysis of internal validity (EFA and CFA) and external validity. Measurement invariance across gender, age, and ethnicity was measured in 271 patients supplying information at time 1 and time 2. Of the full sample, 106 patients supplied information at all 5 possible time points and comprised the sample for analysis of change in overall scores and subscales over the course of treatment. At each analytic stage, the largest possible sample was used, based on the availability of relevant patient record information.

### Measures

Details of Natsal-SF Clinical (from this point on, NSFC) and NSOG items with response distributions are provided in Tables 1–2. For NSFC items on sexual problems, the original questionnaire contained measures of both frequency and distress. As exploratory analyses indicated that distress items provided a better model fit than frequency items, we selected these (with the exception of the item on anxiety, where the frequency measure was selected to aid interpretability). NSOG items are shown using the original 11-point response scale. To align NSOG items with the 4- and 5-point NSFC scales, the main analysis used NSOG items rescaled as follows: 0/1 = 1, 2/3 = 2, 4/6 = 3, 7/8 = 4, 9/10 = 5. Supplementary analyses indicated no loss of information using this approach.

Patient records also contained information on gender, age, ethnicity, sexual orientation, clinician diagnosis, depression using the PHQ-9 (Arroll et al., 2010), anxiety using the GAD7 (Spitzer et al., 2006), alcohol use disorder using the AUDIT (Babor et al., 2001) and non-prescription drug use; all measured at the initial assessment only. Data from the initial patient assessment were used for the main analysis of internal consistency and external validity, restricting the sample to patients completing both the Natsal-SF Clinical and NSOG measures.

### Analysis

#### Internal Validity and Measurement Invariance

Based on the full sample (*n* = 486), exploratory factor analysis (EFA) using Maximum Likelihood (ML) with varimax rotation was used to determine the number of underlying factors represented by the combined set of 23 items (NSFC and NSOG). Indicator items with low loadings (<.3) on all factors were removed, as were items with cross-loadings. We also removed items loading on a factor that contained fewer than three such items (even if loadings were >0.3). This is because a minimum of three items is needed to identify a latent construct corresponding to a subscale (Koran, 2020). Confirmatory factor analysis (CFA) confirmed that a four-factor solution had the best fit. The four factors were as follows: problem distress; partner relationship; overall sex life; sexual confidence. Table 5 shows the final structure and factor loadings (item loadings on each factor are shown in Supplementary Table S1). In subsequent confirmatory factor analysis (CFA; Table 5), indicator cutoffs applied to assess absolute model fit were >.95 for the Confirmatory Fit Index (CFI) and Tucker-Lewis Index (TLI), <.06 for the root mean square error of approximation (RMSEA) and <.08 for the standardized root mean residual (SRMR; Hu & Bentler, 1999). The CFA confirming good model fit allowed extension to a second-order CFA to confirm the presence of a higher-order factor and permitted testing of measurement invariance as detailed below.

Measurement invariance with respect to gender, age (under 30 years vs. 30 years or more) and ethnicity (white vs. ethnic minority) and time was assessed. Across time, the interval between the first and third measurement points was selected as the maximum interval with a sufficiently large sample size (*n* = 193). Across longer intervals (e.g., comparing time 1 with time 4 or 5), sample sizes for repeated measures did not permit model convergence. Configural invariance (similar factor structure), metric invariance (similar factor loadings), and scalar invariance (similar intercepts and factor loadings) across gender, age, and ethnicity were assessed using multigroup models. For configural invariance, we assessed the absolute fit of a multigroup model with no equality constraints. For metric and scalar invariance, we compared the fit of nested constrained and unconstrained models (Putnick & Bornstein, 2016). Configural, metric, and scalar invariance across time was assessed using longitudinal models (Widaman et al., 2010). Following recommended practice, we used multiple indicators to assess invariance, including differences in model chi-square, CFI, RMSEA, and SRMR (Putnick & Bornstein, 2016). Target cutoffs for changes in model fit statistics between constrained and unconstrained models were set at −0.01 for CFI, 0.01 for RMSEA and 0.015 for SRMR, although there is currently little consensus on these (Putnick & Bornstein, 2016).

Analyses all used Mplus Version 8.1 (Muthén & Muthén, Muthén and Muthén, 1998/2017), with missing information handled using the Full Information Maximum Likelihood method.

#### External Validity

Two procedures were used. First, to assess the validity of individual patient reported problems, we examined bivariate associations between NSFC items on individual problems and clinician diagnoses recorded at the initial assessment where this information was available (*n* = 415). Although the SFEQ measure overall assesses more than sexual problems, this information can help establish the potential clinical utility of the measure.

Second, to assess the validity of the final measure, we examined its associations with patient depression (PHQ-9 cutoff score of 10), anxiety (moderate and severe, cut-off scores, respectively, 10 and 15 on GAD7), alcohol use in excess of recommended guidelines (AUDIT C-score 4+ for men, 3+ for women) and non-prescription drug use using logistic regression. Anxiety is strongly implicated in sexual dysfunction (Barlow, 1986) and there is strong evidence that depression/anxiety and sexual dysfunction can be comorbid (Laurent & Simons, 2009). Alcohol and recreational drug use are obviously implicated in Substance/Medication-Induced Sexual Dysfunction, and alcohol can be a factor in erectile difficulties and low desire (American Psychiatric Association, 2013). Few community studies report on alcohol and recreational drug use as risk factors, although one key study found evidence of association between specific difficulties and alcohol or recreational drug use in community populations (Johnson et al., 2004). Based on existing evidence, we would expect a reasonably high correlation between depression and anxiety and a less strong correlation between alcohol and recreational drug use. Note that, as a study taking place in a real-world clinic, choice of external variables was restricted to data routinely collected from patients.

#### Responsiveness to Change

Change in factor scores for the combined measure, and for subscales over a course of five therapy sessions, was explored among patients for whom data were available at all five time points (*n* = 106). This was the maximum number of sessions for which sufficient patient data were collected to permit subgroup analyses. We calculated effect sizes by dividing the change in score over time by the standard deviation of the change score. As a study based on existing clinical data, we did not have a control group (patients who did not receive therapy). Observing change per se may not be helpful since even if the change is in the expected direction it may be unclear what is being picked up (De Vet et al., 2011). To assess whether the change was meaningful, we thus undertook two different sensitivity analyses. These sought to test whether differences in the magnitude of change were as expected across subgroups of patients and domains of sexual function. We expected to observe the following differences:

(1) Change with therapy may be more pronounced among younger patients since they are less likely to have physical health comorbidities (Field et al., 2013). It is also plausible that young people are less likely to have attitudes toward sex and experiences of sexual problems that have become entrenched over a significant period of time.
(2) Therapeutic intervention should produce more change in issues closely related to individual confidence and distressing feelings about specific problems. This is partly because the biopsychosocial model gives primacy to patient subjective perceptions of their experiences and typically seeks to support them in understanding the problem and changing how they respond to their experiences (Brotto & Basson, 2014). In contrast, broader subjective assessments of overall sex life (e.g., satisfaction) are influenced by a wide range of factors (including the presence or absence of a partner), and relational function is partly dependent on the behavior of another (Hewison et al., 2017). Each of these might be less amenable to change.

All analyses used Stata version 16.0.

## Results

Information on NSFC and NSOG items was collected from 486 patients at the first appointment. Table 3 shows sample characteristics. The full sample (*n* = 486) comprised 45.9% female; 62.1% white; and 41.6% were under 30, 46.7% were 30–49, and 11.7% were 50 or over. Clinic data on sexual orientation were incomplete (43.4% missing). Table 4 shows primary clinician diagnoses according to patient gender. For men, the most common primary clinician diagnoses were erectile problems (erectile “difficulties”/“disorder”/“dysfunction,” *n* = 137, 52%), early ejaculation (*n* = 47, 18%), and hyposexual desire disorder (*n* = 21, 8%). For women, the most common primary clinician diagnoses were genito-pelvic pain/penetration disorder (*n* = 131, 59%), anorgasmia (*n*= 25, 11%) and sexual interest/arousal disorder (SIAD) (*n*= 14, 6%). The Natsal-SF Clinical asks about eight sexual problems. Among those reporting any problem “sometimes,” “often” or “very often,” there was a median of four problems per patient (data not shown).

### Structural Validity and Measurement Invariance

Staged EFA suggested a four-factor structure. Ten items performed poorly due to low loadings, insufficient number of items per factor or cross-loadings. These were NSFC items on anxiety, pain, premature orgasm, erectile problems (men)/vaginal dryness vaginal dryness (women), partner’s sexual difficulties; and NSOG items on importance of sex life, severity of sexual problem, distress over sexual problem, quality of sex life and satisfaction with sex life. The five items from the NSOG and the NSFC items on anxiety and partner’s sexual difficulties were all discarded. The remaining items – pain, premature orgasm, erectile problems (men)/vaginal dryness (women) – were individually not well aligned with one another within this clinic population. However, the extent of distress from whichever of these specific problems was most burdensome was sufficiently well aligned with the other less specific problems (lack of enjoyment, no interest in sex, no excitement) to warrant inclusion in the final subscale called “problem distress.” Thus, the NSFC items on pain, premature orgasm, erectile problems (men)/vaginal dryness (women) were retained for patient completion, and a new item (most distress caused by any of these three problems) was created to contribute to the overall score. This allowed us to retain information on specific problems.

A four-factor structure was supported by the CFA, with factors representing problem distress, partner relationship, sex life, and sexual confidence. A second-order CFA model confirmed that all four factors loaded on to a single underlying factor. Model fit was satisfactory (CFI = 0.940, TLI = 0.924, SRMR = 0.054, RMSEA = 0.059 with *p* ≤ .05 equal to 0.085). Table 5 shows the factor structure and loadings for the combined NSFC/NSOG instrument.

Configural invariance of the four-factor structure was satisfactory: across gender, age, ethnic group, and time, CFI was 0.92–0.93, RMSEA 0.05–0.07, and SRMR was <0.08 for all models. Metric invariance was confirmed across gender, age group, ethnic group, and time for all indices of model fit. Scalar invariance received partial confirmation: although differences in model chi-square were statistically significant for all groups, one or more differences in CFI, RMSEA, and SRMR fell within the cutoffs applied. (For further details of tests for measurement invariance, see Supplemental File Table S1.)

### External Validity

To establish the validity of patient reports of specific problem distress, we explored associations between relevant SFEQ items and common clinician diagnoses. Patient reports of distress were generally in agreement with clinician diagnoses. For example, 88% of men diagnosed with erectile problems reported being fairly or very distressed by erectile problems, compared to only 23% of men diagnosed with hyposexual desire disorder. Similarly, 92% of women diagnosed with anorgasmia reported being fairly or very distressed by inability to climax, compared to 36% of women diagnosed with genitopelvic pain/penetration disorder. (Further details provided in the Supplemental File, Table S2.)

To establish the validity of the overall score, we used information supplied by patients on mental health and health behaviors. Of the full sample of 486 at first clinic appointment, 222 patients supplied information on depression, 217 on anxiety, 222 on alcohol use, and 234 on non-prescription drug use. Controlling for patient gender and age, combined NSFC/NSOG factor scores were associated with a greater likelihood of moderate and severe anxiety and of depression (Table 6). These associations were all in the expected direction, confirming external validity. As expected, the combined measure was not associated with alcohol or non-prescription drug use (not shown).

### Responsiveness to Change

There were 106 patients (male *n* = 52; female *n* = 54) who completed the NSFC and NSOG instruments across a sequence of five therapy sessions. As shown in Table 7, the combined measure detected an improvement in scores across both younger (18–29 year old) and older (30–74 year old) age groups (mean change –0.35 (SD 0.47) for 18–29 year olds and mean change –0.17 (0.40) for patients aged 30–74).

**Figure 1:**
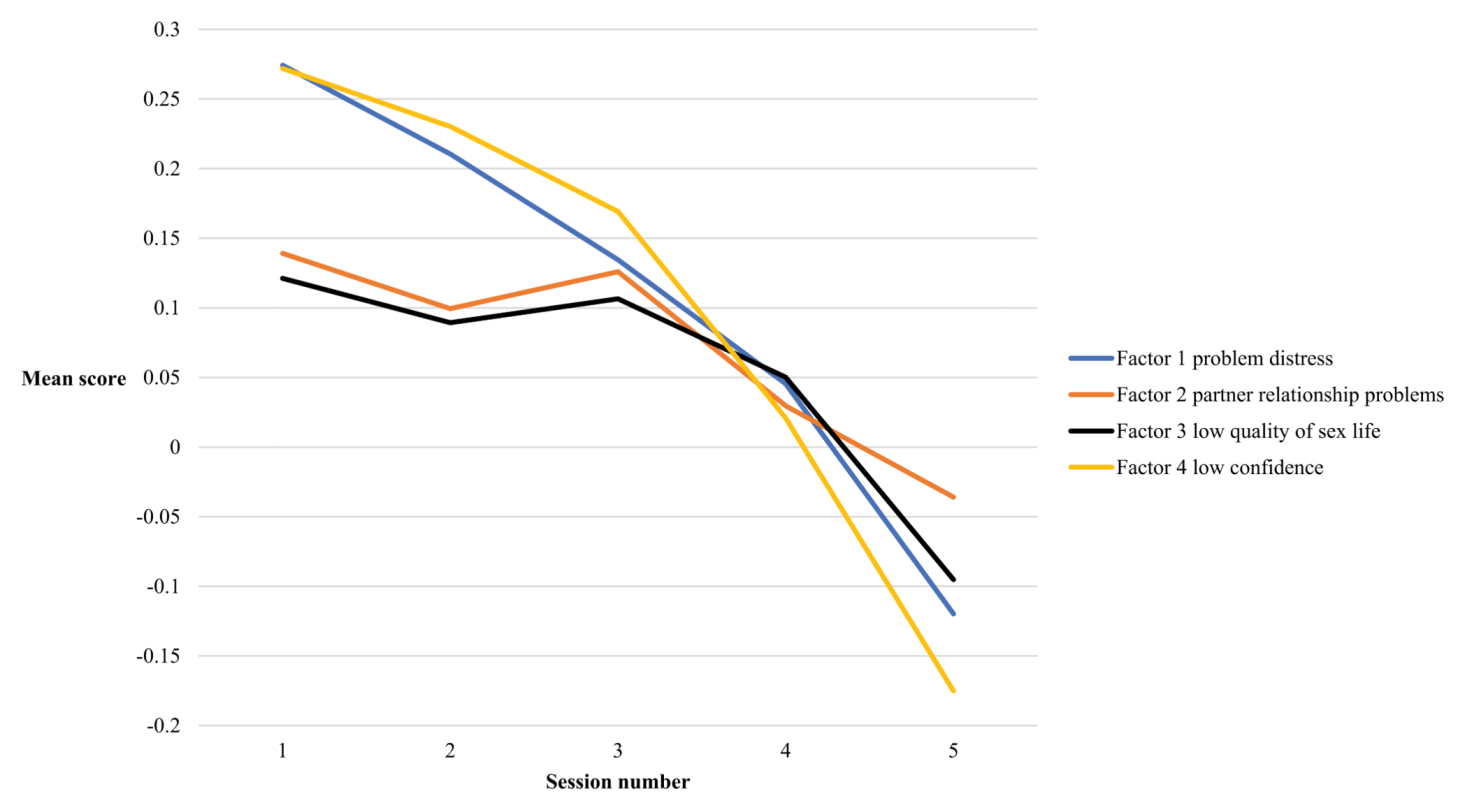
Change in SFEQ subscales over five sessions, *n* = 106.

As expected, effect sizes were larger among younger patients (effect size 0.7 for 18–29 year olds; and 0.4 for 30–74 year olds). Effect sizes were also larger for the problem distress and confidence subscales than for the partner relationship and sex life subscales: among 18–29 year olds effect size for problem distress was 0.7 and sexual confidence was 0.9 compared with 0.4 for partner relationship and 0.4 for sex life (Table 7 and Figure 1). Thus, our second expected difference was confirmed in the data. Confirmation of both these expected differences across patient groups and subscales suggests that the change detected by the SFEQ is meaningful.

## Discussion

The SFEQ is a brief (16-item) clinical measure that captures the effects of biopsychosocial intervention on sexual problem distress, overall quality of sex life, partner relationships and sexual confidence. The SFEQ was designed by combining the best-performing items from two recently developed clinical measures: the Natsal-SF Clinical (15 items) and the NSOG1 (3 items). It has a good model fit (internal validity) and is associated with anxiety and depression (external validity). Measurement invariance (configural and metric) was established across gender, age, ethnicity, and time. We found some evidence for scalar invariance, although there is no established consensus regarding appropriate cutoffs for differences in model fit (Putnick & Bornstein, 2016). By combining measurement of specific problems with “softer” therapy goals, such as confidence, satisfaction, and improved sexual relationship, we believe the SFEQ is uniquely placed as an evaluation tool for holistic approaches to addressing sexual problems.

In addition to brevity and simplicity, the SFEQ is straight-forward for patients to understand and complete; suitable for all genders, all sexual orientations and for different age and ethnic groups. Four of the items on specific problem distress are combined, to give a 13-item measure for scoring purposes. A note on scoring is provided in the Supplementary File.

A possible limitation of the SFEQ is the lack of detailed information on specific sexual problems, although for clinic patients this is likely to be compensated for by clinician notes. It is important to note that the SFEQ is an evaluation tool, not a diagnostic one. Although a strength of the SFEQ is the inclusion of questions about the sexual relationship context, this adds complication to scoring, since these items are not applicable to patients with no regular sexual partner in the last month. In this study, our statistical software imputed missing data, but this technique may not always be available. Further work is required to establish an easy-to-use scoring system that does not rely on imputation.

The SFEQ has a number of strengths and limitations with regard to gender and sexual identity. The male and female versions of the SFEQ are identical except for an item on vaginal dryness (female version) and erectile difficulties (male version). The benefits of this include ease of comparison in effectiveness trials, and simplicity in monitoring and audit of clinic work. It also raises the possibility of utility in couple therapy in which sexual function is a key focus, alongside measures such as the 28-item Golombok Rust Inventory of Marital State (Rust & Golombok, 2010) and generic Clinical Outcomes Routine Evaluation – Outcome Measure therapy outcome measure (Evans et al., 2002). In theory, the measure should be relevant to anyone with a vagina or a penis, regardless of their gender identity, but testing for validity in trans populations was not possible in this study and should be a focus for future work, particularly given the paucity of measures relevant to this group. Similarly, due to missing data, we are limited in exploring the suitability of the measure across patients according to sexual identity and gender(s) of sexual partners, although the measure can be completed irrespective of these. Given the known variation in reporting of distressing sexual problems by sexual orientation and partner gender (Waterhouse & Burkill, 2019), this should be another focus for future research. As a trade-off for brevity, there are aspects of sexual functioning that may be addressed in the course of psychosexual therapy but are not assessed in the SFEQ. This includes things like sexual communication skills, acceptance of sexuality and satisfaction with treatment.

A limitation of the study is that we were unable to assess test–retest reliability, although this has already been established for items included in the Natsal-SF in a community sample (Mitchell et al., 2012). This study used already-collected clinical monitoring data and the retrospective design meant that it was not possible to correlate the SFEQ against widely used, more specialized measures of sexual function (Tabatabaie, 2014). Clinician diagnoses did not indicate the severity of the problem, so we were unable to use these as a measure of validity for the whole measure. Nonetheless, we did find that diagnoses correlated reasonably with patient-reported problems, especially given that exact correspondence is not to be expected (King et al., 2007). The measure was validated in a multidisciplinary service including medical and psychosexual interventions. Due to the nature of the combined assessment model (psychological and pharmacological), it is not possible to establish which aspects of the assessment or intervention the effects noted were linked to, and therefore which were responsible for the largest effects. Future evaluation work employing the SFEQ may be able to tease out these effects. A further limitation is that the clinic was primarily able to offer interventions for erectile difficulties and genital pelvic pain penetration disorder. This meant that other sexual problems – such as SIAD (sexual interest/arousal disorder) – are under-represented in the patient group on which the SFEQ has been validated.

Measures such as the SFEQ are critical to efforts to establish the effectiveness of biopsychosocial treatment models. This, in turn, is vital to attract funding and political support for service provision. Clinically significant sexual problems affect a sizable minority of people; in Britain, around 4% of men and women aged 16–74 report sexual problems meeting clinical severity criteria, only a third of whom have sought professional help (Mitchell et al., 2016). Studies suggest that far more people would like professional help than actually receive it (Dunn et al., 2017). Although this is partly due to individual beliefs about sex and the appropriateness of seeking medical help (Mitchell et al., 2016; Moreira et al., 2005), it is also true that free or affordable therapeutic clinics are scarce in most countries. Sexual problems, despite their prevalence and impact on mental and relational well-being, receive scant attention from policymakers (Mitchell et al., 2013; Population Health Directorate, 2019), and public services are chronically under-funded (Davis, 2019). Our hope is that measures such as the SFEQ can be used to demonstrate the effectiveness of the approach and therefore support clinicians to attract funding for services and to work collaboratively across disciplines (e.g., urology, clinical psychology, and gynecology).

In conclusion, we believe the SFEQ meets a critical gap in measurement tools to assess the effectiveness of biopsychosocial treatment. As a free-to-use, noncommercial measure, we intend it to be accessible to publicly funded services providing holistic treatment for sexual problems.

## Supplementary Material

Supplemental Material

## Figures and Tables

**Table 1:** Distribution of responses, Natsal-SF clinical version (*n* = 486).

Sexual problems			Frequency (%)		
					Always/did not have sex last month due to this
	Never	Not very often	Sometimes	Very often
1) Felt anxious during sex*	11.2	12.8	24.4	26.0	25.6
	Not a problem	Not at all distressed	Distress A little distressed	Fairly distressed	very distressed
2) Lacked interest in having sex	24.0	12.0	21.9	26.7	15.4
3) Lacked enjoyment in having sex	23.5	9.1	22.6	26.5	18.4
4) Felt physical pain as a result of sex	41.8	11.0	12.4	15.0	19.8
5) Felt no excitement or arousal during sex	28.5	11.2	20.2	23.8	16.4
6) Did not reach a climax	28.1	14.3	21.7	19.8	16.2
7) Reached a climax more quickly than would like	51.2	14.9	13.6	8.9	11.4
8) Erectile problems (men)/dry vagina(women)	21.5	9.3	15.8	23.9	29.6
**Sexual relationship with partner**	Agree strongly	Agree	Neither agree nor disagree	Disagree	Disagree strongly
9) My partner and I share about the same level of interest in having sex	11.8	20.0	14.5	34.9	18.8

10) My partner and I share the same sexual likes and dislikes	13.7	39.5	27.7	16.0	3.1
11) My partner has experienced sexual difficulties in the last month	10.6	16.9	11.0	28.7	32.7
	Always	Most of the time	Sometimes	Not very often	Hardly ever
12) Feel emotionally close to partner when engage in sex together	35.7	39.1	16.8	5.5	2.9
**Perceptions of sex life**	Agree strongly	Agree	Neither agree nor disagree	Disagree	Disagree strongly
13) I feel satisfied with my sex life	2.6	6.0	15.9	42.8	32.8
14) I feel distressed or worried about my sex life	32.8	47.5	10.6	5.3	3.8
15) I have avoided sex because of sexual difficulties (my own or partner’s)	27.2	35.3	13.9	14.8	8.8

These distributions exclude missing information. There was missing information for 11–14% of NSFC problem distress items, 47–51% of NSFC partner relationship items (these were only applicable to patients having a regular sexual partner for the past month), 3–4% of NSFC sex life items and 1% of NSOG items.

* Because anxiety and distress overlap conceptually, frequency rather than distress was used to score severity on this item.

**Table 2:** Distribution of responses NSOG items (*n* = 486).

	11-point response scale (%)										
	0	1	2	3	4	5	6	7	8	9	10
1) How important is your sex life to you?	Not important 1.0	0.2	1.4	1.7	2.1	3.3	3.5	17.1	22.7	14.4	Important 32.6
2) How severe is your sexual problem?	Not severe 0.8	0.6	2.7	3.3	2.7	10.2	8.7	21.4	19.1	12.5	Severe 17.9
3) What is the quality of your sexual life?	Poor 16.2	7.1	11.9	17.5	9.2	10.6	12.3	6.2	5.0	2.1	Good 2.1
4) How sexually confident are you?	Not confident 11.6	5.6	9.5	13.9	10.2	13.7	11.8	9.8	6.6	3.5	Confident 3.7
5) How well does your body work sexually?	Not well 11.3	6.7	13.5	15.6	10.6	11.7	8.5	9.4	6.7	2.9	Well 3.1
6) How satisfied are you with your sex life?	Not satisfied 22.2	10.6	16.6	13.5	9.1	10.4	8.5	4.4	2.1	1.5	Satisfied 1.2
7) How distressed are you by your sexual problem?	Not distressed 0.8	1.7	5.2	4.6	4.3	5.6	8.7	15.3	19.2	15.1	Distressed 19.6
8) How confident do you feel engaging in sexual activity?	Not confident 11.2	6.9	13.1	10.8	10.8	12.1	11.0	10.2	4.8	4.8	Confident 4.4

For the main analyses, NSOG item responses were combined as follows: 0/1 = 1, 2/3 = 2, 4/6 = 3, 7/8 = 4, 9/10 = 5. These distributions exclude missing information. There was missing information for 1% of NSOG items.

**Table 3:** Sample information (*n* = 486).

		*n*	%
Gender	Female	223	45.9
	Male	263	54.1
Ethnic group	White	302	62.1
	Indian/Pakistani/Bangladeshi	23	4.7
	Black/Caribbean/African	41	8.4
	Mixed/Other	47	9.7
	Asian	14	2.9
	Missing	59	12.1
Age (years)	<20	8	1.7
	20–29	194	39.9
	30–39	161	33.1
	40–49	66	13.6
	50–59	41	8.4
	60+	16	3.3
Sexual orientation	Bisexual	10	2.1
	Heterosexual	265	54.5
	Missing	211	43.4

**Table 4:** Primary clinician diagnosis by patient gender*.

		n	%
Men	Erectile difficulties/disorder/dysfunction	137	52
	Hyposexual desire disorder	21	8
	Early ejaculation	47	18
	Delayed ejaculation	20	8
	Partner has sexual difficulties	8	3
	No diagnosis	30	11
Women	Genito-pelvic pain/penetration disorder	131	59
	Anorgasmia	25	11
	Sexual interest/arousal disorder	14	6
	Partner has sexual difficulties	11	5
	Anaerobic bacterial vaginosis	1	0
	No diagnosis	41	18

* The distribution of diagnoses in part reflects that the clinic was primarily able to offer treatment for Erectile Difficulties (men) and Genito-Pelvic Pain/Penetration Disorder (women).

**Table 5:** Factor structure and loadings for a combined NSFC/NSOG instrument derived from confirmatory factor analysis (analytic sample = 486).

Factor	Item*	Factor loading
**Factor 1: Problem distress**	Distress due to no interest in sex	0.75
	Distress due to no enjoyment of sex	0.78
(NATSAL Q2,3,5 plus 6, 7, 4, 8; Table 1)	Distress due to no excitement/arousal	0.75
	Distress from no climax; climax quicker than would like; pain; or erectile difficulties/vaginal dryness (whichever causes the most distress)	0.50
**Factor 2: Partner relationship**	share same level of interest in sex	0.76
	share same sexual likes and dislikes	0.63
(NATSAL Q9,10,12; Table 1)	feel emotionally close to partner during sex	0.40
**Factor 3: Overall sex life**	Satisfied with sex life	0.66
	Distressed by sex life	−0.62
(NATSAL Q13,14,15; Table 1)	Avoided sex	−0.73
**Factor 4: Sexual confidence**	Sexually confident	0.82
	How well body works well sexually	0.64
(NSOG Q4,5,8; Table 2)	Confident engaging in sexual activity	0.88
**Overall sexual function**	Factor 1	0.59
	Factor 2	0.54
	Factor 3	0.90
	Factor 4	−0.69

Higher scores for factors 1, 2, and 3, and lower scores for factor 4, denote lower sexual function. In the overall measure, higher scores denote lower function.*

**Table 6:** Associations between combined measure and patient anxiety, depression, and substance use.

	Outcome (sample prevalence %)					
	Moderate anxiety (*n* = 217) (31%)		Severe anxiety (*n* = 217) (13%)		Depression (n = 222) (26%)	
	OR (95% CI)	*p*	OR (95% CI)	*p*	OR (95% CI)	*p*
Combined NSFC/NSOG	2.29 (1.20–4.40)	.012	7.17 (2.46–20.90)	<.001	2.05 (1.04–4.03)	.037
*Sample size*	*217*		*217*		*222*	

Moderate anxiety: GAD7 score 10+, Severe anxiety GAD7 score 15+, depression PHQ9 score 10+. Models were all adjusted for gender and age. OR = odds ratio, CI = confidence interval, *p* = probability

**Table 7:** Mean change in the overall and subscale scores over five therapy sessions, among patients in different age groups.

	Age 18–29 (*n* = 45)			Age 30–74 (*n* = 61)		
	gMean change (SD)	*p*-value	Effect size	Mean change (SD)	*p*-value	Effect size
Problem distress	−0.59 (0.84)	<.001	0.7	−0.25 (0.83)	.021	0.3
Partner relationship	−0.23 (0.53)	.006	0.4	−0.14 (0.56)	.063	0.2
Sex life	−0.31 (0.72)	.005	0.4	−0.14 (0.50)	.028	0.3
Sexual confidence	−0.62 (0.66)	<.001	0.9	−0.32 (0.67)	<.001	0.5
Overall measure	−0.35 (0.47)	<.001	0.7	−0.17 (0.40)	.002	0.4
