# Supplementary material for: The Sexual Function Evaluation Questionnaire (SFEQ) to Evaluate Effectiveness of Treatment for Sexual Difficulties: Development and Validation in a Clinical Sample
Source: J Sex Res. Author manuscript; Available in PMC 2024 Dec 8. (PMC7616988; doi:10.1080/00224499.2021.1986800)
Supplement: Supplemental Material [file EMS157716-supplement-Supplemental_Material.zip › Supplemental File validation and scoring.docx]

**Supplemental File**

**Table S1** *Tests of Measurement Invariance*

|  |  | Chi-square | df | CFI | RMSEA | (90% CI) | SRMR | Chi-square diff | df difference | p-value | delta CFI | delta RMSEA | delta SRMR |
| --- | --- | --- | --- | --- | --- | --- | --- | --- | --- | --- | --- | --- | --- |
| **Gender (males n=262, females n=223)** | | | |  |  |  |  |  |  |  | cutoff 0.01 | cutoff 0.01 | cut off 0.015 |
| Configural |  | 258.152 | 118 | 0.920 | 0.074 | 0.058-0.082 | 0.074 |  |  |  |  |  |  |
|  |  |  |  |  |  |  |  |  |  |  |  |  |  |
| Metric | Constrained | 313.709 | 144 | 0.904 | 0.070 | 0.059-0.080 | 0.088 |  |  |  |  |  |  |
|  | Loadings vary | 299.386 | 131 | 0.904 | 0.073 | 0.062-0.084 | 0.083 | 14.323 | 13 | 0.351 | 0.000 | -0.003 | 0.005 |
|  |  |  |  |  |  |  |  |  |  |  |  |  |  |
| Scalar | Constrained | 313.709 | 144 | 0.904 | 0.070 | 0.059-0.080 | 0.088 | 55.557 | 26 | 0.001 | -0.016 | -0.004 | 0.014 |
|  | Loadings and intercepts vary | 258.152 | 118 | 0.920 | 0.074 | 0.058-0.082 | 0.074 |  |  |  |  |  |  |
|  |  |  |  |  |  |  |  |  |  |  |  |  |  |
| **Ethnic (white n=301, minority n=125)** | | |  |  |  |  |  |  |  |  |  |  |  |
| Configural |  | 216.960 | 118 | 0.930 | 0.063 | 0.049-0.076 | 0.067 |  |  |  |  |  |  |
|  |  |  |  |  |  |  |  |  |  |  |  |  |  |
| Metric | Constrained | 267.514 | 144 | 0.913 | 0.063 | 0.052-0.075 | 0.090 |  |  |  |  |  |  |
|  | Loadings vary | 251.014 | 131 | 0.915 | 0.066 | 0.053-0.078 | 0.075 | 16.500 | 13 | 0.223 | -0.002 | -0.003 | 0.015 |
|  |  |  |  |  |  |  |  |  |  |  |  |  |  |
| Scalar | Constrained | 267.514 | 144 | 0.913 | 0.063 | 0.052-0.075 | 0.090 |  |  |  |  |  |  |
|  | Loadings and intercepts vary | 216.960 | 118 | 0.930 | 0.063 | 0.049-0.076 | 0.067 | 50.554 | 26 | 0.003 | -0.017 | 0.000 | 0.023 |
|  |  |  |  |  |  |  |  |  |  |  |  |  |  |
| **Age (<30 years n=202, 30+ years n=283)** | | | |  |  |  |  |  |  |  |  |  |  |
| Configural |  | 228.996 | 118 | 0.936 | 0.062 | 0.050- 0.074 | 0.063 |  |  |  |  |  |  |
|  |  |  |  |  |  |  |  |  |  |  |  |  |  |
| Metric | Constrained | 277.632 | 144 | 0.923 | 0.062 | 0.051-0.073 | 0.076 |  |  |  |  |  |  |
|  | Loadings vary | 272.946 | 131 | 0.918 | 0.067 | 0.056-0.078 | 0.072 | 4.686 | 13 | 0.182 | 0.005 | -0.005 | 0.004 |
|  |  |  |  |  |  |  |  |  |  |  |  |  |  |
| Scalar | Constrained | 293.213 | 144 | 0.916 | 0.065 | 0.055-0.076 | 0.076 |  |  |  |  |  |  |
|  | Loadings and intercepts vary | 228.996 | 118 | 0.936 | 0.062 | 0.050- 0.074 | 0.063 | 48.636 | 26 | 0.014 | -0.020 | 0.003 | 0.013 |
|  |  |  |  |  |  |  |  |  |  |  |  |  |  |
| **Time (time 1 n=193, time 2 n=271)** | | |  |  |  |  |  |  |  |  |  |  |  |
| Configural |  | 517.618 | 308 | 0.915 | 0.050 | 0.043-0.058 | 0.079 |  |  |  |  |  |  |
|  |  |  |  |  |  |  |  |  |  |  |  |  |  |
| Metric | Constrained | 566.871 | 332 | 0.905 | 0.051 | 0.044-0.058 | 0.080 |  |  |  |  |  |  |
|  | Loadings vary | 553.641 | 322 | 0.907 | 0.052 | 0.044-0.059 | 0.081 | 13.230 | 10 | 0.211 | 0.002 | 0.001 | 0.001 |
|  |  |  |  |  |  |  |  |  |  |  |  |  |  |
| Scalar | Constrained | 566.871 | 332 | 0.905 | 0.051 | 0.044-0.058 | 0.080 |  |  |  |  |  |  |
|  | Loadings and intercepts vary | 517.618 | 308 | 0.915 | 0.050 | 0.043-0.058 | 0.079 | 49.253 | 24 | 0.002 | 0.010 | -0.001 | -0.001 |
|  |  |  |  |  |  |  |  |  |  |  |  |  |  |

Note: Figures in RED show where difference between model fit of constrained and unconstrained models is statistically significant (Chi-square) or exceeds recommended thresholds for change in other model fit statistics. Figures in GREEN show where difference is not significant (Chi-square) or within recommended thresholds for change in other model fit statistics (Putnick & Bornstein, 2016)

**Reference**

Putnick, D. L., & Bornstein, M. H. (2016). Measurement Invariance Conventions and Reporting: The State of the Art and Future Directions for Psychological Research. *Developmental review : DR, 41*, 71-90. doi:10.1016/j.dr.2016.06.004

**Table S2** *Associations between clinician diagnoses and patient reports of problem distress*

|  | **Clinician diagnoses** | | | | | |  |
| --- | --- | --- | --- | --- | --- | --- | --- |
|  | **Men** | | |  | **Women** | |  |
|  | **Early ejaculation** | **Erectile problems** | **Hyposexual desire disorder** |  | **Genito-pelvic pain/ penetration disorder** | **Anorgasmia** |  |
| *n* | *47* | *137* | *21* |  | *131* | *25* |  |
| **Patient report: "fairly distressed" or "very distressed" by** | % | % | % | *p* | % | % | *p* |
| No interest in sex | 17 | 39 | 76 | <.001 | 42 | 52 | 0.634 |
| No enjoyment from sex | 24 | 33 | 60 | 0.091 | 53 | 67 | 0.420 |
| Painful sex | 5 | 6 | 6 | 0.414 | 82 | 28 | <.001 |
| No excitement from sex | 15 | 39 | 67 | <.001 | 40 | 75 | 0.007 |
| No orgasm | 5 | 30 | 28 | 0.001 | 36 | 92 | <.001 |
| Premature orgasm | 81 | 23 | 42 | <.001 | 3 | 5 | 0.033 |
| Erectile problems | 23 | 88 | 61 | <.001 | N/A | N/A |  |
| Vaginal dryness | N/A | N/A | N/A |  | 45 | 38 | 0.689 |

Note: Analysis for each sex was restricted to diagnoses reported by > 20 patients. *p*-value from Chi-square tests of association between patient report and clinician diagnosis.

**A note on scoring the SFEQ**

Section A – In part A of items, the response “No” (ie did not experience problem) is scored as 0, response “did not have sex because of (specific problem) is scored as a 4 (equivalent to "very distressed") and response “did not have sex, but for a different reason” is coded as missing.

Section A requires a single new item: "problem causing the most distress" to be derived from  items 4,6,7 and 8 - using the item with the highest score.

Computing the final score: it is suggested that individual items are weighted using the factor loadings shown in Table 5 of the main manuscript. In cases where Section B is not applicable, use of imputation software to supply missing information is advised.
