# Supplementary material for: The Sexual Function Evaluation Questionnaire (SFEQ) to Evaluate Effectiveness of Treatment for Sexual Difficulties: Development and Validation in a Clinical Sample
Source: J Sex Res. Author manuscript; Available in PMC 2024 Dec 8. (PMC7616988; doi:10.1080/00224499.2021.1986800)
Supplement: Supplemental Material [file EMS157716-supplement-Supplemental_Material.zip › Supplementary File -Factor-item loadings SFEQ.docx]

**Supplementary Table 1: Factor Item loadings for items on the SFEQ (four factor solution based on Confirmatory Factor Analysis)**

| EFA 4-factor solution |  |  |  |  |
| --- | --- | --- | --- | --- |
|  | Rotated varimax loadings | | | |
|  | Factor 1: Problem distress | Factor 2: Partner relationship | Factor 3: Overall sex life | Factor 4: Sexual confidence |
| SFEQ Item |  |  |  |  |
| Distress due to no interest in sex | **0.652** | 0.269 | 0.211 | 0.130 |
| Distress due to no enjoyment of sex | **0.770** | 0.123 | 0.135 | 0.083 |
| Distress due to no excitement in sex | **0.712** | 0.209 | 0.043 | 0.185 |
| Distress from no orgasm, premature orgasm, pain, or erectile difficulties/vaginal dryness (whichever causes the most distress) | **0.516** | -0.215 | 0.218 | 0.173 |
| Share similar level of interest in sex | 0.104 | **0.703** | 0.230 | 0.107 |
| Share same sexual likes and dislikes | 0.052 | **0.633** | 0.074 | 0.063 |
| Feel emotionally close to partner during sex | 0.073 | **0.401** | -0.008 | 0.057 |
| Satisfied with sex life | 0.156 | 0.236 | **0.461** | 0.331 |
| Distressed by sex life | -0.165 | -0.009 | **-0.690** | -0.119 |
| Avoided sex | -0.162 | -0.170 | **-0.657** | -0.239 |
| Sexually confident | -0.129 | -0.144 | -0.111 | **-0.822** |
| Body works well sexually | -0.264 | 0.009 | -0.194 | **-0.587** |
| Confident engaging in sexual activity | -0.105 | -0.168 | -0.263 | **-0.795** |
